# Indexing grazing-incidence X-ray diffraction patterns of thin films: lattices of higher symmetry

**DOI:** 10.1107/S1600576719003029

**Published:** 2019-04-01

**Authors:** Josef Simbrunner, Sebastian Hofer, Benedikt Schrode, Yves Garmshausen, Stefan Hecht, Roland Resel, Ingo Salzmann

**Affiliations:** a Department of Neuroradiology, Vascular and Interventional Radiology, Medical University Graz, Auenbruggerplatz 9, Graz, 8036, Austria; b Institute of Solid State Physics, Technical University Graz, Petersgasse 16, Graz, 8010, Austria; c Department of Chemistry and IRIS Adlershof, Humboldt-Universität zu Berlin, Brook-Taylor-Strasse 2, Berlin, 12489, Germany; d Department of Physics, Department of Chemistry and Biochemistry, Centre for Research in Molecular Modeling (CERMM), Centre for Nanoscience Research (CeNSR), Concordia University, 7141 Sherbrooke Street W., SP 265-20, Montreal, Quebec, Canada H4B 1R6

**Keywords:** grazing incidence X-ray diffraction, indexing, fibre texture, uni-planar texture, mathematical crystallography

## Abstract

Grazing-incidence X-ray diffraction studies on organic thin films are often performed on systems showing fibre-textured growth. However, indexing their experimental diffraction patterns is generally challenging, especially if low-symmetry lattices are involved. Recently, analytical mathematical expressions for indexing experimental diffraction patterns of triclinic lattices were provided. In the present work, the corresponding formalism for crystal lattices of higher symmetry is given and procedures for applying these equations for indexing experimental data are described. Two examples are presented to demonstrate the feasibility of the indexing method. For layered crystals of the prototypical organic semiconductors di­indeno­perylene and (*ortho*-di­fluoro)­sexi­phenyl, as grown on highly oriented pyrolytic graphite, their yet unknown unit-cell parameters are determined and their crystallographic lattices are identified as monoclinic and orthorhombic, respectively.

## Abstract 2

A general indexing procedure for grazing-incidence X-ray diffraction patterns recorded from fibre-textured films is introduced for lattices of monoclinic and higher-symmetry systems. The analytical mathematical expressions are given and their use is demonstrated in detail for two cases (monoclinic and orthorhombic).

## 1. Introduction   

**Figure 1:**
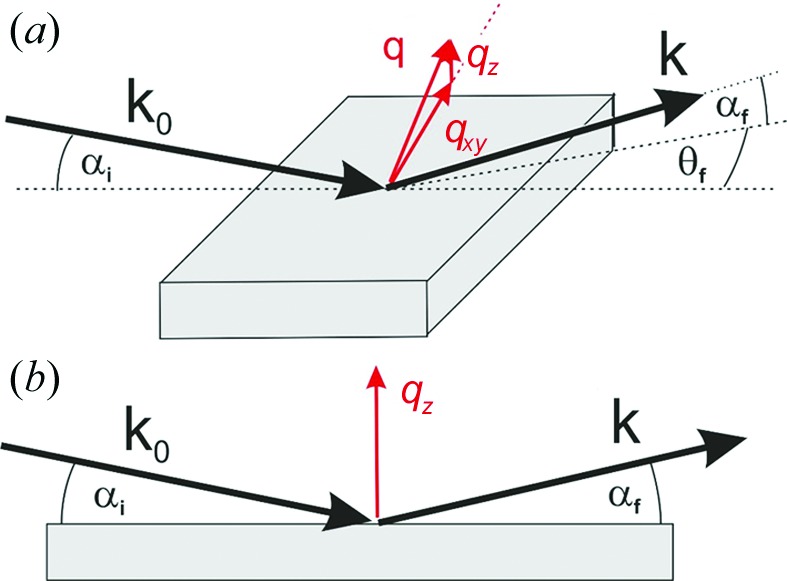
X-ray diffraction geometries for crystal structure investigations of thin films, with **k**_0_ and **k** as the wavevectors of the incident and scattered X-ray beams, respectively; α_i_ is the angle of incidence, α_f_ the out-of-plane scattering angle and θ_f_ the in-plane scattering angle. (*a*) Grazing-incidence X-ray diffraction, where the scattering vector is split into an in-plane component *q_xy_* and an out-of-plane component *q_z_*. (*b*) Specular X-ray diffraction in co-planar geometry, with α_i_ = α_f_ and θ_f_ = 0, where the scattering vector **q** consists only of a nonzero out-of-plane component *q_z_*.

Crystalline thin films are often characterized by grazing-incidence X-ray diffraction (GIXD) owing to the high surface sensitivity of the technique. A schematic drawing of the experimental method is shown in Fig. 1 ▸(*a*). To achieve this surface sensitivity, the angle of incidence of the primary beam relative to the sample surface (α_i_) is chosen close to the critical angle of total external reflection, and the scattered X-ray beam encloses an in-plane angle θ_f_, as well as an out-of-plane angle α_f_ relative to the surface. The primary X-ray beam, described by the wavevector **k**_0_, and the scattered beam **k** determine the scattering vector **q** by **q** = **k** − **k**_0_. In this geometry the in-plane component *q_xy_* and the out-of-plane component *q_z_* of **q** are given by
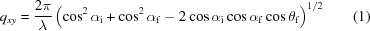
and
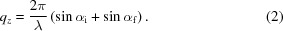



A key direction in reciprocal space is the *z* direction at *q_xy_* = 0, which reveals the orientation of crystals relative to the substrate surface. In the important case of crystalline fibre-textured films, it defines the crystallographic plane of the thin-film crystallites which is parallel to the substrate surface (*i.e.* the contact plane). However, in GIXD geometry the *z* direction is inaccessible in reciprocal space. To experimentally assess this direction, one needs to resort to specular X-ray diffraction [*cf*. Fig. 1 ▸(*b*)], where the incidence angle of the primary beam (α_i_) and the exit angle of the scattered X-ray beam (α_f_) are equal. This results in a scattering vector **q** always perpendicular to the substrate surface and the scattering vector then exclusively has a *q_z_* component (with *q_xy_* = 0). In the following, a diffraction peak observed via specular X-ray diffraction is denoted as 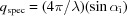. The correlation between the scattering vector **q** and the corresponding lattice periodicities is obtained via the Laue equation: for the appearance of a diffraction peak the scattering vector **q** has to be equal to a reciprocal lattice vector **g**.

In general, indexing of GIXD patterns means assigning Laue indices to the observed Bragg peaks. In the specific case of GIXD data recorded on fibre-textured films, which is covered in the present manuscript, two components of the reciprocal lattice vectors – *q_z_* and *q_xy_* – are taken from experimental reciprocal-space maps and available for indexing (Smilgies & Blasini, 2007 ▸; Hailey *et al.*, 2014 ▸). As the fibre-textured crystals have a well defined crystallographic contact plane, the rotation matrix of the crystallites relative to the sample surface must be considered (Shmueli, 2006 ▸), *i.e.* the contact plane of the investigated crystals must be determined. In many cases, it can be deduced from specular X-ray diffraction data; however, the correct assignment to a crystallographic plane with Miller indices *u*, *v* and *w* cannot be obtained from specular X-ray diffraction alone, if the crystal structure is unknown (Salzmann & Resel, 2004 ▸; Smilgies & Blasini, 2007 ▸; Hailey *et al.*, 2014 ▸; Jiang, 2015 ▸). By combining the peak positions of the GIXD pattern (*q_xy_*, *q_z_*) with the specular peak position (*q*_spec_), mathematical expressions can be derived where the required number of unknown parameters for indexing the pattern is significantly reduced. In recent work, we demonstrated this approach for the general case of the triclinic crystallographic system (Simbrunner *et al.*, 2018 ▸).

Here, we now turn our focus to crystallographic lattices of higher symmetry. Obviously, the equations for the in-plane and out-of-plane components of the reciprocal-space vectors become less complex if the number of unit-cell parameters is reduced. This can facilitate the indexing procedure; however, the parameters of the rotation matrix do not reduce. In the following, we first concentrate on monoclinic systems and subsequently present mathematical expressions for all other systems. Via two practical examples, we then explicitly demonstrate the application of these equations. We stress that our indexing procedure does not rely on knowing either the chemical structure of the material or the experimental intensities of the diffraction peaks. We show that the presented approach can succeed with a limited number of reflections for experimental data of high quality.

## 2. Indexing method   

For the mathematical treatise a laboratory coordinate system is assumed, where the *xy* plane runs parallel to the substrate surface; *a*, *b*, *c*, α, β and γ are the parameters of the (direct) unit cell, and a*, b*, c*, α*, β* and γ* are the reciprocal cell parameters (Giacovazzo, 2011 ▸), which are summarized in Table 1 ▸. If the (001) lattice plane is parallel to the substrate surface in a GIXD experiment, the reciprocal lattice vector **g** with its Laue indices *h*, *k* and *l* can be represented by the equation
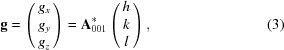
where the matrix 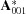 is given as
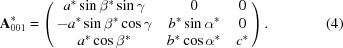
When the Laue condition **q** = **g** is fulfilled, diffraction can be observed.

If the (001) lattice plane is not parallel to the substrate surface, the matrix 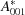 must be transformed, *i.e.* the rotation matrix of the thin-film crystallites relative to the substrate surface has to be considered (Shmueli, 2006 ▸). In particular, it has to be rotated around the zone axis which is defined by the (001) plane and the new contact plane (*uvw*). Then the reciprocal-space vector with the Laue/Miller indices *u*, *v* and *w*, **g**_*uvw*_, is perpendicular to the contact plane. Its magnitude is purely out of plane and corresponds to the specular scan, *g*_spec_. Furthermore, the out-of-plane component *g_z_* and in-plane component *g_xy_* of any reciprocal-space vector **g**_*hkl*_ can be determined by their scalar product and vector (cross) product with **g**_*uvw*_, respectively. As the magnitude of a vector and the scalar product of two vectors are both rotation invariant, the unrotated expressions, *i.e.* equation (3), for **g**_*hkl*_ and **g**_*uvw*_ are sufficient. Therefore, the following expressions result:
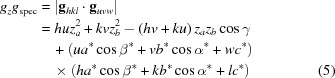
with 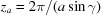, 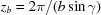,
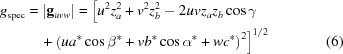
and
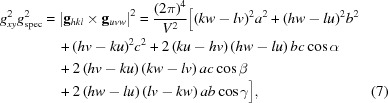
where the volume is
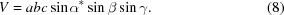



Furthermore, from equation (3) the length of the vector 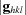, *g_xyz_*, can be determined as
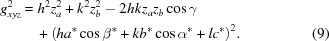



Using equations (5) and (6), equation (9) can be rewritten as
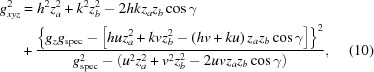
and by algebraic transformations the following expression can be derived:
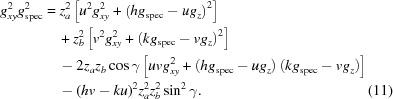



As a result of symmetry relationships, further analogous expressions for the cell parameter triples (*a*, *c*, β) and (*b*, *c*, *α*) and their corresponding Miller and Laue indices can be derived.

From equations (5) and (6) the following expression can be obtained:
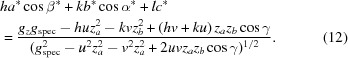



From equation (12), the residual unit-cell parameters *c*, α, β and the Laue indices *l* can be determined (Truger *et al.*, 2016 ▸). Analogously, the following equation is valid:
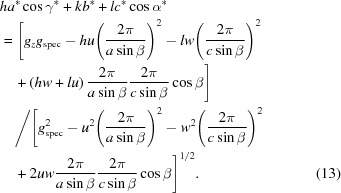



In a monoclinic system (α = γ = 90°), equation (11) reduces to
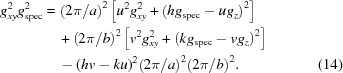



Equation (14) comprises – in addition to the rotation parameters *u* and *v* – only the lattice parameters *a* and *b*, as well as the Laue indices *h* and *k*. This facilitates the mathematical analysis, where the integer variables can be varied and only two real unknowns must be calculated.

In rare cases, if the net planes oriented parallel to the substrate surface have a weak structure factor, which inhibits the acquisition of specular diffraction data (Djuric *et al.*, 2012 ▸), *g*_spec_ must be treated as an additional real unknown parameter, which necessitates a more extensive numerical analysis. In the most general case, if no contact plane exists, *u*, *v* and *w* are non-integers. In this case it is preferable to use an alternative notation of the rotation matrix (see Appendix *A* [App appa]).

For calculating the remaining unit-cell parameters *c*, β and the Laue indices *l*, the following expression results from equation (12):
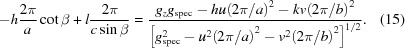
Analogously to equation (14), by choosing related substitutions on the basis of the symmetric properties of the relations for *g_xyz_*, *g_xy_*, *g_z_* and *g*_spec_ for the general (triclinic) case, the following expressions hold:
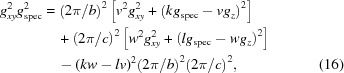


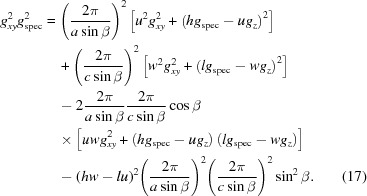
Furthermore, equation (13) reduces to
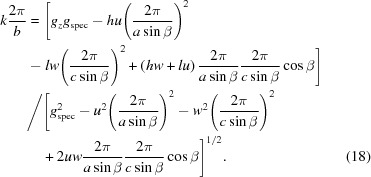
For orthorhombic systems, the formulae are valid with β = 90°. In Table 2 ▸ we summarize the mathematical expressions for *g_xyz_*, *g_z_*, *g_xy_* and the volume *V* in triclinic, monoclinic and orthorhombic systems. In tetragonal and cubic lattices, in addition to β = 90°, *a* = *b* and *a* = *b* = *c*, respectively. In Appendix *B* [App appb] useful formulae for tetragonal, trigonal and hexagonal systems are listed.

As every linear combination of the unit-cell vectors obeys the Laue condition, the mathematical solutions are not unique and include superlattices (Santoro *et al.*, 1980 ▸). The definite crystallographic solution, the reduced cell, is defined as the cell that satisfies the conditions derived from the reduction theory of quadratic forms (Niggli, 1928 ▸). The main conditions for reduction require that the unit cell is based on the three shortest vectors of the lattice; such a unit cell is then called a Buerger cell (Buerger, 1957 ▸). However, this cell may not be unique. An unambiguous unit cell is the ‘reduced cell’ defined by Niggli; the criteria are listed in detail in *International Tables for Crystallography* (De Wolff, 2016 ▸). In general, the criteria for reduced cells demand that 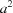 ≤ 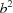 ≤ 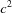 and that the angles are either acute (type I) or obtuse (type II). Geometrical ambiguities, called lattice metric singularities, which generate subcells of lower symmetry, may occur in some indexing cases (Santoro & Mighell, 1970 ▸; Mighell, 2000 ▸). Such singularities can often be easily detected from simple relationships between the lattice parameters of the two cells (Boultif & Louër, 2004 ▸).

In the following we turn to employing the formalism derived above to determine the unit-cell parameters of two prototypical organic semiconductors, which show as yet unknown polymorphs if grown in thin films on substrates of highly oriented pyrolytic graphite (HOPG).

## 3. Examples   

### 3.1. Experiments   

Thin films of di­indeno­perylene (DIP, C_32_H_16_) and of *ortho*-di­fluoro­sexi­phenyl (*o*-F_2_-6P, C_36_F_2_H_24_) were grown on freshly cleaved HOPG (ZYA quality) by physical vapour deposition in a high vacuum (final nominal film thickness 30 nm; base pressure < 5 × 10^−6^ Pa). The films were characterized at beamline W1 at the synchrotron radiation facility DORIS (HASYLAB, Hamburg, Germany). GIXD experiments were performed together with specular X-ray diffraction using a goniometer in pseudo 2 + 2 geometry and a one-dimensional detector (MYTHEN, Dectris). The wavelengths of the primary radiation were 1.1796 and 1.1801 Å for DIP and *o*-F_2_-6P, respectively. GIXD experiments were performed using incident angles of the primary beam relative to the HOPG substrate of α_i_ = 0.13 and 0.15° for DIP and *o*-F_2_-6P, respectively. Reciprocal-space maps were recorded by keeping the sample fixed and by performing a series of detector scans along the in-plane scattering angle θ_f_ at differently fixed out-of-plane scattering angles α_f_. The vertical mounting of the detector allows the simultaneous measurement of Δα_f_ = 3.5°. The diffraction pattern was transformed to reciprocal space using the custom-made software *PyGID* (Moser, 2012 ▸). The resulting reciprocal-space maps give the measured intensities on a logarithmic scale by a colour code; the exact positions of the Bragg peaks in terms of *q_xy_* and *q_z_* were determined by Gaussian fits. The *q_z_* values of the peak positions were corrected for refraction effects; maximum variations of 0.011 and 0.014 Å^−1^ are obtained for DIP and *o*-F_2_-6P, respectively (Resel *et al.*, 2016 ▸).

### 3.2. Di­indeno­perylene   

**Figure 2:**
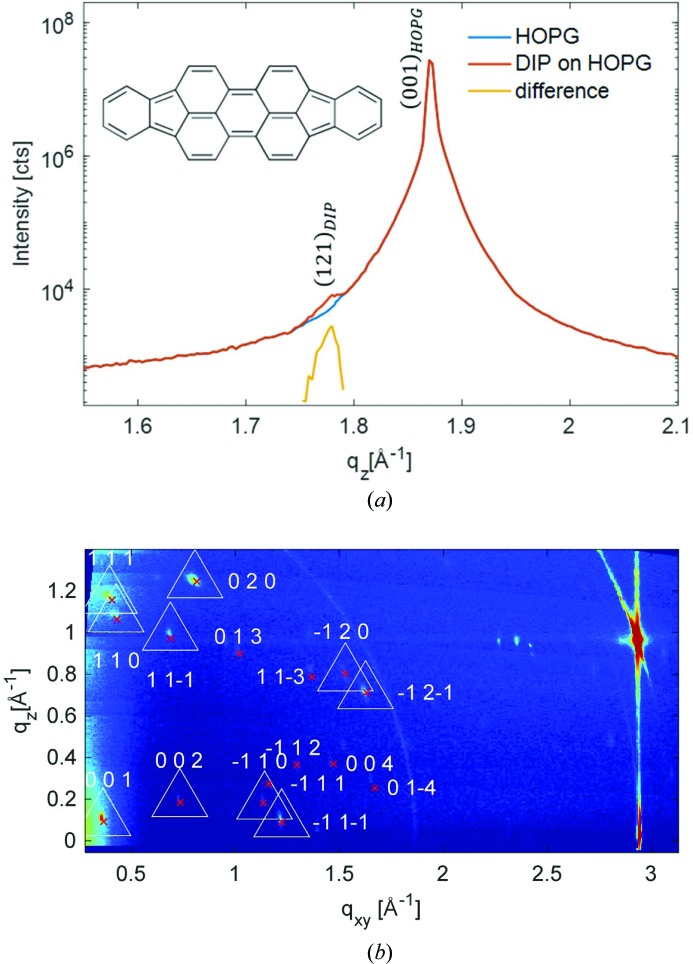
X-ray diffraction of DIP grown on a (001) substrate of HOPG. The chemical structure of the molecule is given as an inset. (*a*) Specular diffraction pattern of the HOPG substrate with and without DIP crystals, as well as the difference between the two patterns around the specular diffraction peak of DIP crystals. (*b*) Reciprocal-space map measured by grazing-incidence X-ray diffraction with a selection of peaks which were used for indexing. The centres of the triangles and the crosses give the experimental and calculated peak positions, respectively; the respective Laue indices are also given.

A specular scan of DIP crystals grown on a HOPG substrate is shown in Fig. 2 ▸(*a*). The dominant diffraction peak at *q_z_* = 1.872 Å^−1^ (*d* = 3.356 Å) can be identified as the 002 peak of the HOPG substrate. Forming the difference with the reference data recorded for the plain substrate (orange curve) reveals a clear diffraction peak at *q*_spec_ = 1.776 Å^−1^, which is assigned to the DIP adsorbate and corresponds to a lattice spacing of 3.54 Å. Note that a specular reflection from DIP at the same position has been observed before for gold substrates by Dürr *et al.* (2003 ▸). The authors concluded on a lying molecular orientation from their data and denoted this polymorph as the λ phase; however, no direct determination of the unit cell by indexing GIXD reciprocal-space-map data has been performed before. Our reciprocal-space map of DIP on HOPG is shown in Fig. 2 ▸(*b*). Strong diffraction features of the HOPG substrate are located along *q_xy_* = 2.94 Å^−1^. Additionally, weak diffraction features at *q_xy_* = 2.24 Å^−1^/*q_z_* = 0.98 Å^−1^ and at *q_xy_* = 2.34 Å^−1^/*q_z_* = 0.98 Å^−1^ are also due to the HOPG substrate. Bragg peaks at low *q* values (*q* ^2^ = *q_xy_* ^2^ + *q_z_* ^2^) were selected for the indexing procedure and lie at centres of the triangles in Fig. 2 ▸(*b*). In total, we only used ten Bragg peaks (based on their *q_xy_* and *q_z_* positions) together with the Bragg peak of the specular diffraction pattern (*q*_spec_) for our indexing procedure. Note that *q*_spec_, *q_xy_* and *q_z_* are experimental results which describe specific components of the scattering vector, while *g*_spec_, *g_xy_* and *g_z_* refer to these components within the reciprocal lattice. According to the Laue condition, the individual components of the scattering vector **q** and of the reciprocal lattice vectors **g** must be equal.

As known crystal structures of DIP exhibit a monoclinic lattice (Heinrich *et al.*, 2007 ▸; Kowarik *et al.*, 2009 ▸), in a first step of indexing, equation (14) may be chosen. The combination of Bragg peaks from the reciprocal-space map (*q_xy_* and *q_z_*) with the peak from the specular scan (*q*_spec_) reduces the number of parameters considerably to two integer numbers (instead of three) for the Miller indices of the contact plane, to two integer numbers (instead of three) for the Laue indices of each Bragg peak, and to two real numbers (instead of four) for the lattice constants. In theory, by using a quadratic equation, *a* and *b* can be calculated from two independent pairs of (*g_xy_*, *g_z_*) (see Appendix *C* [App appc]). Because of experimental inaccuracies, however, we prefer treating equation (14) as linear with the unknowns 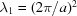, 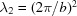 and 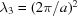
 × 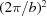. The first step of indexing is then based on a systematic variation of integer variables: (i) a pair of Miller indices for the contact plane (the crystallographic plane of DIP crystals parallel to the substrate surface), and (ii) two Laue indices for each of the selected Bragg peaks from the reciprocal-space map [Fig. 2 ▸(*b*)]. By using several pairs of (*g_xy_*, *g_z_*), sets of overdetermined linear equations are then obtained, from which the solutions with the least errors *e* concerning the system of equations (*i.e.* norm of the residuals) and the factor 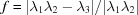 are selected. Furthermore, the volume of the resulting unit cell should be as small as possible. From λ_1_, λ_2_ and λ_3_ the solution for *a* and *b* can be optimized (see Appendix *D* [App appd]). In our case, for *u* = 1 and *v* = 2, *e* and *f* of the optimal solution are 4.8 × 10^−3^ and 1.1 × 10^−3^, respectively. The next best solutions to the optimal choice of the Laue indices *h* and *k* have almost the same error *e* (plus 1% and minus 1%, respectively) but a larger factor *f* of about 40 and 56%, respectively.

As equation (16) is analogous to equation (14), two solutions (for *a* and *c*, respectively) with consistent unit-cell parameter *b*, Miller index *v* and Laue indices *k* must exist. If the Miller indices are chosen to be *v* = 2 and *w* = 1, an appropriate solution can be found. Thus, a monoclinic lattice with the contact plane (121) results and the cell parameters *a*, *b* and *c* and the Laue indices *h*, *k* and *l* can be assigned to the experimental pattern. In a last step, using equation (15), the last cell parameter β can be obtained.

Alternatively, in a first step of indexing, equation (17), which is valid for any lattice type, can be used. For this procedure we use the following specific algorithm: appropriate mathematical substitutions transform equation (17) into linear equations with three real unknowns, containing the lattice constants *a*, *c* and β (see Appendix *E* [App appe]). Assigning two low integers as the Miller indices *u* and *w*, and choosing Laue indices *h* and *l* in a specific range, is a starting point to find solutions for the lattice constants *a*, *c* and β. In theory, a set of lattice constants can be obtained from three equations with independent pairs of *g_xy_* and *g_z_*. Being linearized, these equations can be solved analytically by determining the inverse matrix of their coefficients. To account for experimental inaccuracies, however, all ten peaks can be included simultaneously, again for creating an overdetermined system of linear equations. This system can be solved either by multiplying both sides of the equations with the transpose of the coefficient matrix or by QR decomposition to solve the least-squares problem (Bronshtein *et al.*, 2015 ▸). In addition to an optimized approximated solution of the unknown variables, the associated error can be calculated. By varying the integer variables, the final solution to the overdetermined system is found. This is done by choosing the optimal set of equations regarding a small error and a volume of the resulting unit cell that is as small as possible. To overcome the computational complexity with an increasing number of equations, this procedure is performed stepwise. In a first step, four equations with small *q* values are chosen, for which the possible range of Laue indices is restricted. Only systems with errors not exceeding a certain limit are included further. Thus the Laue indices *h* and *l* for all reflections can be assigned and the unit-cell parameters *a*, *c* and β are calculated. A modification of this algorithm relies on comparing the systematically varied coefficients of the ten linear equations and seeking clusters of related coefficients.

Thus, for *u* = 1 and *w* = 1 a solution for a first set of lattice constants (*a*, *c*, β) and the Laue indices *h* and *k* can be obtained. Then, in a second step, it can be checked if equation (18) results for all peak positions (*q_xy_, q_z_*) in integer multiples of 2π/*b*. If – as is the case here – this condition is fulfilled, the assumption of a monoclinic lattice is justified. Furthermore, using equation (13) for a triclinic system and calculating α and γ does not result in a more accurate final solution. Consequently, the lattice constant *b* is obtained and the Laue indices *k* for all reflections can be assigned.

In a last step, when all integer variables have been assigned, the values of the real lattice parameters can be numerically fitted. For this procedure, the expressions for *g_xyz_*, *g_xy_* and *g_z_* in Table 2 ▸ can be used. For our example of DIP on HOPG, we then obtain the following solution, which obeys the scalar product criteria for type-II cells: *u* = 1, *v* = 2, *w* = 1; *a* = 7.149, *b* = 8.465, *c* = 16.62 Å, α = 90, β = 93.14, γ = 90°, *V* = 1004.5 Å^3^. The accuracy of the result can be assessed by the factors 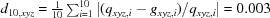 and 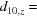 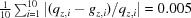, where 10 is the number of reflections, and (*q*_*xyz*,*i*_, *q*_*z*,*i*_) are the measured and (*g*_*xyz*,*i*_, *g*_*z*,*i*_) the calculated peak positions of the *i*th reflection. As an example, the influences of the refraction correction on the lattice constants are demonstrated by using uncorrected values for *q_z_*. In this case, we would then obtain significantly different lattice parameters instead: *a* = 7.194, *b* = 8.440, *c* = 16.60 Å, α = 90, β = 93.54, γ = 90°, *V* = 1005.7 Å^3^ (*d*_10,*xyz*_ = 0.006, *d*_10,*z*_ = 0.025).

Importantly, however, as the underlying equations do not allow a unique mathematical solution, it must still be checked if the lattice obtained corresponds to the reduced unit cell. For this purpose, three reciprocal lattice vectors **g**_1_, **g**_2_ and **g**_3_, *e.g.* of the three independent Laue triples (0, 0, 1), (1, 1, 0) and (−1, 1, 0), are calculated and the matrix
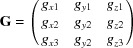
is formed. By multiplying its inverse matrix **G** ^−1^ with vectors 2π(*m*_1_, *m*_2_, *m*_3_)^T^, where *m_i_* are systematically varied integers in a reasonable range (*e.g*. between −3 and 3), lattice vectors of the unit cell and its superlattices can be obtained (Simbrunner *et al.*, 2018 ▸). Listing the lengths of these vectors in ascending order yields 7.149, 8.465, 11.08, 14.30, 16.616 and 16.62 for the six shortest vectors. But as the first five vectors are coplanar, our solution matches the Buerger and reduced cell.

On the basis of this solution, peak positions are calculated and plotted in Fig. 2 ▸(*b*) as crosses. In addition to the ten peaks we initially selected, all other observed peaks can now be indexed according to this unit cell.

Our result is significantly different from the known crystal structures of DIP, that is, an enantiotropic polymorph that is stable at temperatures above 423 K (Heinrich *et al.*, 2007 ▸) showing lattice constants of *a* = 7.171, *b* = 8.55, *c* = 16.80 Å, α = 90, β = 92.42, γ = 90°, *V* = 1029.0 Å^3^, as well as a thin-film phase grown on sapphire (Kowarik *et al.*, 2009 ▸) with *a* = 7.09 ± 0.04, *b* = 8.67 ± 0.04, *c* = 16.9 ± 0.5 Å, α = 90, β = 92.2 ± 0.2, γ = 90°, *V* = 1037 ± 30 Å^3^. In Table 3 ▸ we summarize the various results. Comparing these cells highlights that DIP grows on HOPG in a previously unknown polymorph, most likely in the same polymorph which has been referred to as λ phase in the literature before (Dürr *et al.*, 2003 ▸; Kowarik *et al.*, 2006 ▸; Casu *et al.*, 2008 ▸).

### 3.3. *ortho*-Di­fluoro­sexi­phenyl   

**Figure 3:**
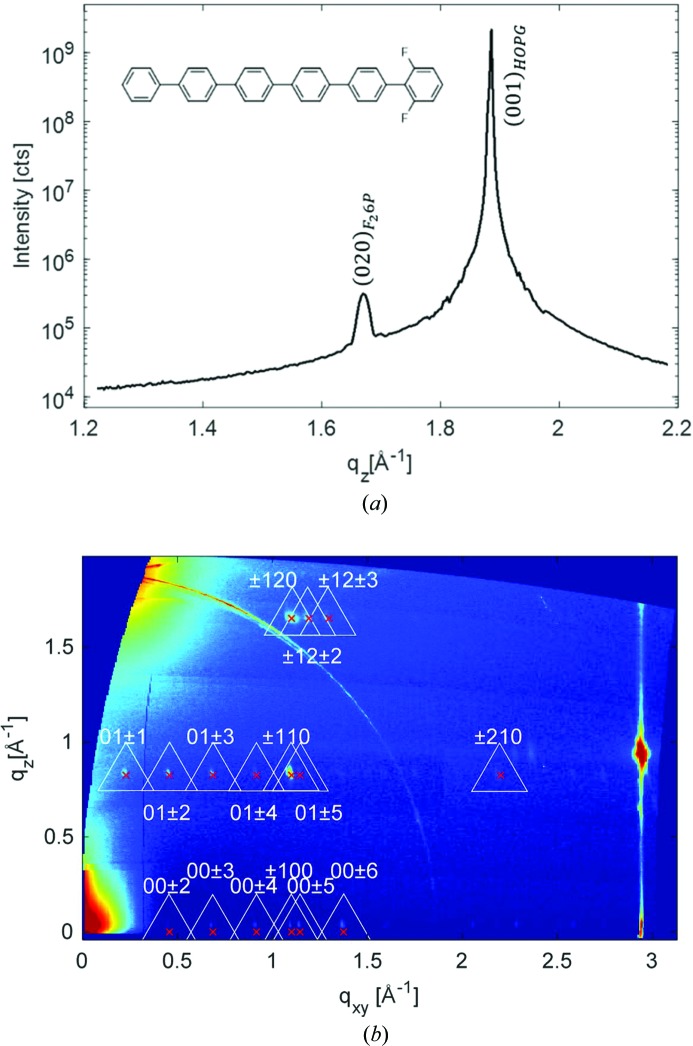
X-ray diffraction of *o*-F_2_-6P, as grown on a (001) plane of HOPG; the inset gives the chemical structure of the molecule. (*a*) Specular diffraction pattern of the sample with the characteristic diffraction peak of the HOPG substrate and of the *o*-F_2_-6P crystals. (*b*) Reciprocal-space map measured by grazing-incidence X-ray diffraction. Selected peaks for the indexing procedure are marked by triangles around the maximum of the diffracted intensity; the crosses give the calculated peak positions based on indexing together with the assigned Laue indices.

The experimental GIXD data for the *o*-F_2_-6P film [the synthesis of *o*-F_2_-6P is reported by Niederhausen *et al.* (2018 ▸)] as grown on HOPG are shown in Fig. 3 ▸. We now observe a well pronounced specular diffraction peak at *q*_spec_ = 1.636 Å^−1^, corresponding to a lattice spacing of 3.84 Å in the out-of-plane direction. Like the case of DIP above, this points to lying π-stacked growth of the molecules on the HOPG substrate, a growth behaviour that has been reported before for related systems (Salzmann *et al.*, 2012 ▸). The reciprocal-space map reveals, besides the diffraction peaks of HOPG (see discussion above), a highly regular sequence of Bragg peaks located at constant *q_z_* values. In this example, the indexing procedure was performed on 16 selected reflections.

In marked contrast to non-substituted 6P growing in a monoclinic crystal structure (Baker *et al.*, 1993 ▸; Resel, 2003 ▸), from the highly symmetric diffraction pattern, an ortho­rhombic lattice with its contact plane parallel to the (010) plane (*u* = *w* = 0) can be assumed for *o*-F_2_-6P on HOPG. As for the specular scan the relation 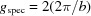 (*v* = 2) holds, the following expressions (see Table 2 ▸) follow:
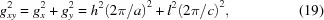


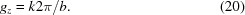



On that basis, we obtained for the unit cell of *o*-F_2_-6P on HOPG: *u* = 0, *v* = 2, *w* = 0; *a* = 5.724, *b* = 7.659, *c* = 27.424 Å, α = β = γ = 90°, *V* = 1202.3 Å^3^. The accuracy of the result can be assessed by 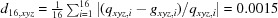 and 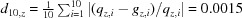, as for *d*_*N*,*z*_ only the 10 reflections with *k* > 0 are included. Note that as a result of equation (24) the Laue indices *h* and *l* can be either positive or negative. Again, if uncorrected values for *q_z_* were used, *b* = 7.614 Å would follow. It can be proven that **a**, **b** and **c** are the shortest non-coplanar unit-cell vectors.

As an illustration of the comparison between calculated peaks and experimental data, the peak positions are plotted in Fig. 3 ▸(*b*) as crosses; there are no crystal structures known so far for molecular crystals of *o*-F_2_-6P. The low out-of-plane lattice spacing that we observe via specular diffraction strongly points to π-stacked growth of this fluorinated 6P derivative, if grown on HOPG. This is remarkable insofar as non-substituted 6P grows in a herringbone arrangement of the molecules in all known structures and on numerous surfaces including HOPG (Resel, 2003 ▸) and, therefore, it is obviously the (one-sided) fluorination of the molecule that changes its growth behaviour dramatically. A similar behaviour has been observed for pentacene (herringbone) and perfluoro­pentacene on HOPG (π-stacking) (Salzmann *et al.*, 2012 ▸), as well as for 6,13-bis(triiso­propyl­silylethynyl)pentacene and pentacene­quinone (Swartz *et al.*, 2005 ▸). The transition from inclined to parallel molecular planes in these structures has been ascribed to the impact of intramolecular polar bonds by the authors. For *o*-F_2_-6P, no bulk crystal structures have been published yet, and structural characterization was limited to sub-monolayers on Ag(111) by low-temperature scanning tunnelling microscopy (Niederhausen *et al.*, 2018 ▸). There, in contrast to non-fluorinated 6P molecules which individually adsorb in the sub-monolayer regime on the metal surface without packing, the authors find a flat lying stack arrangement of the *o*-F_2_-6P molecules with small lateral shifts along the row direction. The net dipole moment of *o*-F_2_-6P is derived as 1.1 Debye owing to the polar C—F bonds at the *ortho* position of the outer phenyl ring, which appear to maximize their distance in neighbouring molecules.

Building upon previous work (Salzmann *et al.*, 2011 ▸; Truger *et al.*, 2016 ▸), the unit cell determined in the present work for *o*-F_2_-6P (and, likewise, for DIP) can now be used to model the molecular arrangement therein, which will be the subject of a forthcoming study.

## 4. Discussion – determining the crystallographic system   

In the case of powder diffraction only the lengths of the reciprocal-space vectors are used for indexing. In the dichotomy method the cell constants are varied in increasingly smaller intervals and the *hkl* indices are subsequently refined using the least-squares method (Boultif & Louër, 1991 ▸, 2004 ▸). The search of indexing solutions typically starts from the cubic end of the symmetry sequence. Each crystal system is explored independently up to a maximum input volume, unless a solution has been found with a higher symmetry.

Indexing of GIXD patterns is based on the knowledge of two components of the reciprocal space vector, the in-plane component *q_xy_* and the out-of-plane component *q_z_* [Fig. 1 ▸(*a*)]. Since the lattice type cannot be assigned *a priori*, we suggest following an iterative approach. As a consequence of imperfect data due to experimental inaccuracies, it seems that starting with a lattice of higher symmetry is favourable, since incoherency becomes evident quickly and the tendency to find only a ‘local minimum’ when finally optimizing the cell parameters increases with the number of variables in the equations. Moreover, boundary conditions and experimental constraints can be included in the indexing procedure with less numerical effort. If no satisfactory solution can be found with a specific lattice type, the next lower symmetry system will then be used.

In single-crystal diffraction, where reciprocal lattice vectors are used for indexing, in a first step the model parameters are refined in a triclinic setting. If possible symmetry elements are detected, cell refinement with symmetry-bases restraints is performed (Sauter *et al.*, 2004 ▸). In the matrix approach to symmetry (Himes & Mighell, 1987 ▸) this is accomplished by 64 symmetry matrices to check if the transformation leads to identity. Furthermore, systems of higher symmetry imply a high impact of symmetry considerations such as diffraction intensities and systematic absences (Hahn, 2006 ▸). Determination of the symmetry profile in crystallographic structures is a persistent challenge (Hicks *et al.*, 2018 ▸) and certainly lies beyond the scope of the present work.

The final goal in crystallographic analysis is the determination of the most fundamental property of the structure – the correct space group. Four steps are necessary to achieve this endeavour (Marsh, 1995 ▸): (i) the derivation of the correct space lattice, *i.e.* the smallest primitive (reduced) unit cell; (ii) the assignment of the correct Laue group on the basis of the symmetry of the diffraction intensities and an initial decision if a structure is centrosymmetric; (iii) the identification of any systematic absences characteristic of translational symmetry elements (glide planes or screw axes); and (iv) the final decision as to whether or not the structure is centrosymmetric. Higher metric symmetry is usually identified by computer programs (Hicks *et al.*, 2018 ▸).

We emphasize that in the present work we focus only on the first point. For determining the lattice parameters and indexing of the diffraction pattern it is appropriate to choose the crystallographic system of the highest order which can be rationally fitted to the measured reflections. However, it is not the shape of the unit cell that determines the lattice type but the symmetry of the diffraction intensities (Marsh, 1995 ▸).

In GIXD the diffraction intensities are influenced by various parameters. This may impede the determination of the correct Laue group. Furthermore, as the in-plane component of the reflections is measured, the Laue indices in lattices of higher symmetry can be ‘degenerate’, *i.e.* they cannot be assigned with positive or negative sign, as is the case in our example of *o*-F_2_-6P. For the reliable identification of systematic absences it is further necessary to obtain a reasonable number of reflections.

## 5. Conclusion   

Indexing of GIXD data of fibre-textured films is important for phase analysis as well as for the identification of new polymorphs. In the present work, we provide a unifying framework for indexing reciprocal-space maps obtained by GIXD for monoclinic lattices and lattices of higher symmetry. Our approach of including the Bragg peak from a specular X-ray diffraction experiment into the mathematical formalism is of considerable help for indexing of GIXD patterns, where the spatial orientation of the unit cell must be considered. Mathematical expressions with a significantly reduced number of unit-cell parameters are derived, which facilitates the computational efforts. For crystallographic lattices of higher symmetry, where the set of unit-cell parameters is reduced, the specular diffraction peak is still important for determining the orientation of the crystallographic unit cell relative to the sample surface. Procedures are described in detail for how to use the derived mathematical expressions. We demonstrate the high value of our approach by successfully applying our formalism for indexing diffraction patterns of two organic semiconductors grown as crystalline thin films on graphite surfaces. We find a monoclinic lattice for di­indeno­perylene and an orthorhombic lattice for *ortho*-di­fluoro­sexi­phenyl, the unit-cell parameters of which were successfully determined following our approach.

## Figures and Tables

**Table 1:** Relations between the parameters of the direct lattice (*a*, *b*, *c*, α, β, γ) and of the reciprocal lattice (a*, b*, c*, α*, β*, γ*) and the volume of the crystallographic unit cell *V* b* (c*), cosβ* (cosγ*), sinβ* (sinγ*) and cosβ (cosγ) can be derived by cyclic rotation of parameters from entries 1–4, respectively, of row 1.

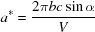	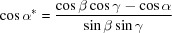	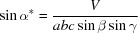	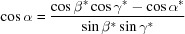
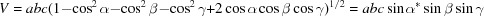			

**Table 2:** Relations for the total length *g_xyz_*, the out-of-plane component *g_z_* and the in-plane component *g_xy_* of the reciprocal-space vectors with indices *hkl* and of the vector *uvw* (*g*_spec_) by using direct and reciprocal lattice parameters and the volume *V* in triclinic, monoclinic and orthorhombic systems

Triclinic
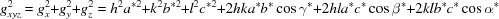
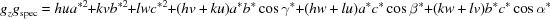
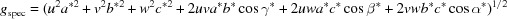
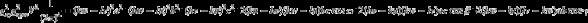
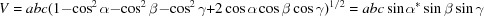

Monoclinic *a* ≠ *b* ≠ *c*, α = γ = 90°
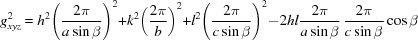
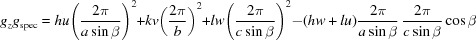
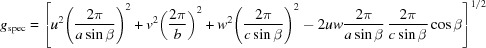
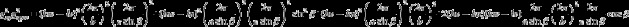
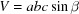

Orthorhombic *a* ≠ *b* ≠ *c*, α =β = γ = 90°
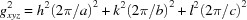
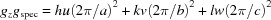
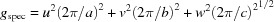
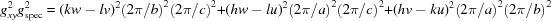
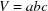

**Table 3:** The lattice constants of di­indeno­perylene at different temperatures compared with our result

	Heinrich *et al.* (2007) ▸	Kowarik *et al.* (2009) ▸	This work
*T* (K)	423	403	300
*a* (Å)	7.1709 (8)	7.09 (4)	7.149 (50)
*b* (Å)	8.5496 (9)	8.67 (4)	8.465 (41)
*c* (Å)	16.7981 (18)	16.9 (5)	16.62 (36)
α (°)	90	90	90
β (°)	92.416 (11)	92.2 (2)	93.14 (93)
γ (°)	90	90	90
Volume (Å^3^)	1028.95	1037	1004.5

**Table 4:** Relations for the total length *g_xyz_*, the out-of-plane component *g_z_* and the in-plane component *g_xy_* of the reciprocal-space vectors with indices *hkl* and of the vector *uvw* (*g*_spec_) and the volume *V* in trigonal and hexagonal crystallographic systems

Trigonal *a* = *b* = *c*, α = β = γ ≠ 90°
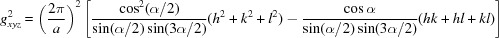
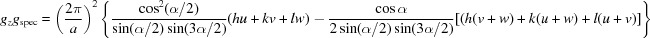
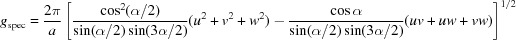
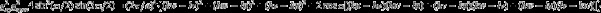
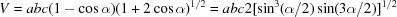

Hexagonal *a* = *b* ≠ *c*, α = β = 90°, γ = 120°
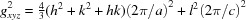
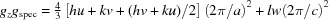
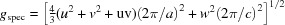
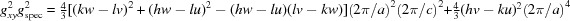
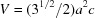

## Appendix A Alternative rotation parameters if no contact plane exists

If *u*, *v* and *w* have to be assumed to be non-integers, it may be preferable to replace the vector **g**_*uvw*_ by a sample surface normal vector **n** = (sinψsinΦ, −cosψsinΦ, cosΦ)^T^ with the rotation angles Φ and ψ. Then for the monoclinic system the following expression can be derived:

The same equation is valid in the orthorhombic case.

For the tetragonal and cubic systems the following expression is valid:

Therefore, especially in the cubic case, it is much easier to solve the equation

first, as in the case of powder diffraction, and then to determine the rotation parameters.

Note that for DIP Φ = 76.11° and ψ = 149.37°. In the case of *o*-F_2_-6P Φ = 90° and ψ = 180°.

## Appendix B Mathematical expressions for tetragonal, trigonal and hexagonal systems

In Table 4 ▸ we summarize the mathematical expressions for *g_xyz_*, *g_z_*, *g_xy_* and the volume *V* in trigonal and hexagonal systems.

From equation (11), the following formulae for  in tetragonal and hexagonal systems, respectively, can be derived:

Tetragonal:

With  and  ×

Hexagonal:

With  and

In both cases, by systematically varying the integer variables *u*, *v*, *h* and *k*, the factor 2π/*a* must be constantly observed for every reflection, allowing the assessment of the appropriate Miller and Laue indices.

## Appendix C Calculating *a* and *b* from two pairs of (*g_xy_*, *g_z_*) in a monoclinic lattice

If (*g*_*xy*,1_, *g*_*z*,1_) and (*g*_*xy*,2_, *g*_*z*,2_) are the components of two Bragg peaks with the corresponding Laue indices (*h*_1_, *k*_1_) and (*h*_2_, *k*_2_), respectively, as a result of equation (14) the following relations arise:

where ,  × ,  and  . Expressing  in equation (28) as a function of  and substituting this term in equation (29) leads to a quadratic equation for , which has the two solutions

where

Consequently,

## Appendix D Determining *a* and *b* from λ_1_, λ_2_ and λ_3_

From λ_1_, λ_2_ and λ_3_ the tentative cell parameters *a*_t_ = 2πλ_1_^−1/2^, *b*_t_ = 2πλ_2_^−1/2^ and their product (*ab*)_t_ = (2π)^2^λ_3_^−1/2^ can be determined. An optimal solution for *a* and *b* can be obtained by minimizing the following function *F*:

This can be achieved if  and  are fulfilled. Then the following solution can be obtained:

where  and     .

## Appendix E Mathematical procedure for analytically determining the cell parameters *a*, *c* and β

For analytically determining the unit-cell parameters *a*, *c* and β from equation (17) it is convenient to introduce the parameters *X_a_*, *X_c_* and *X*_β_ with the substitutional relations

Using these substitutions, equation (17) can be rewritten as

Then from three independent Bragg peak series, the parameters *X_a_*, *X_c_* and *X_β_* can be determined using a system of linear equations. For obtaining *a*, *c* and β from equations (36)–(38) [Disp-formula fd38] the following identity is helpful:
